# Propagation Attenuation Maps Based on Parabolic Equation Method †

**DOI:** 10.3390/s22114063

**Published:** 2022-05-27

**Authors:** Michał Kryk, Krzysztof Malon, Jan M. Kelner

**Affiliations:** Institute of Communications Systems, Faculty of Electronics, Military University of Technology, 00908 Warsaw, Poland; michal.kryk@wat.edu.pl (M.K.); krzysztof.malon@wat.edu.pl (K.M.)

**Keywords:** wireless communications, mobile ad-hoc network (MANET), radio environment map (REM), radio wave propagation, propagation attenuation map (PAM), parabolic equation method (PEM), path loss, terrain topography, digital terrain elevation data (DTED)

## Abstract

Modern wireless communication systems use various technological solutions to increase the efficiency of created radio networks. This efficiency also applies to radio resources. Currently, the utilization of a radio environment map (REM) is one of the directions allowing to improve radio resource management. The REM is increasingly used in emerging mobile ad-hoc networks (MANETs), in particular military tactical networks. In this case, the use of new technologies such as software-defined radio and network, cognitive radio, radio sensing, and building electromagnetic situational awareness made it possible to implement REM in tactical MANETs. Propagation attenuation maps (PAMs) are crucial REM elements that allow for determining the ranges of radio network nodes. In this paper, we present a novel algorithm for PAM based on a parabolic equation method (PEM). The PEM allows determining the signal attenuation along the assumed propagation direction. In this case, we consider terrain topography to obtain a more realistic analysis. Then, we average the adjacent attenuation profiles defined for the selected directions in places where attenuation has not been calculated. To this aim, linear regression is applied. Finally, we define several metrics that allow for the accuracy assessment of determining the PAM as a function of its dimensions.

## 1. Introduction

Currently, we are observing the development and implementation of the fifth-generation (5G) mobile network, i.e., 5G New Radio (NR) [1,2]. At the same time, 5G standards are still being developed for other types of communication systems than terrestrial-wireless, such as satellite [3], wired, optical, hybrid [4], etc. This direction of development is so attractive that attempts are also made to replicate it in military communication systems [5,6,7,8].

A few years ago, the first works and analyses began on the legitimacy of using 5G technologies for defense. 5G technologies are a set of radio and network technologies, i.e., in relation to the physical and higher layers of the ISO open systems interconnection (OSI) reference model, respectively. These technologies often also include technologies that were developed earlier, e.g., with the Long Term Evolution (LTE), LTE-Advance (LTE-A), or LTE-A Pro standards, and which are also implemented in the 5G NR. The most important 5G technologies include massive multiple-input-multiple-output (massive-MIMO), beamforming, millimeter waves, full-duplex communications, software-defined radio (SDR), radio resource management (RRM), dynamic spectrum access (DSA), interference mitigation, energy harvesting communications, ultra-dense network, self-organizing network (SON) [9,10,11,12,13].

In the military sector, work is underway to use the civilian 5G standard for military scenarios. On the other hand, selected 5G technologies are already implemented in the upcoming military systems. While wired and satellite military communications are often based on civilian solutions, wireless military communications, especially at the tactical level, are governed by different rules. In this case, the direct implementation of civil solutions, such as the LTE or 5G NR cellular telephony, is not possible. Tactical military networks are usually classified as mobile ad-hoc networks (MANETs) [14,15]. In this case, there are no fixed base stations, and all network nodes act as mobile stations (MSs). MSs can move at different speeds and in any direction. Hence, the network organization and the coordination of all its elements are challenging.

Some of the aforementioned 5G technologies, such as the RMM, DSA, or SON are already used in military communication systems today, regardless of the used radio resources. At the stage of planning, managing, and coordinating activities of military forces, the so-called battlefield management system (BMS) is used [16,17]. In relation to communication systems, a radio environment map (REM) [18,19] is usually the element of such a BMS. In this case, the REM is responsible for the planning, management, and coordination of the MANET nodes. On the other hand, the REM ensures the implementation of the RMM, DSA, and SON technologies.

Propagation attenuation maps (PAMs) are crucial REM elements that allow for determining the ranges of radio network nodes. The PAMs may base on different propagation models. In this case, the use of statistical propagation models is usually insufficient [20]. Military operations can be conducted in various terrains. It requires that the PAM considers the terrain topography and the different clutter types such as vegetation, buildings, and the ground parameters (i.e., terrain type). In this case, the use of deterministic propagation models is a better solution [20]. This paper focuses on a novel algorithm for determining the PAM. The proposed solution is based on a parabolic equation method (PEM) and linear interpolation. This algorithm is implemented in a REM in the new Polish tactical communication system that used selected 5G technologies. In the next sections, we introduce important aspects related to the military use of 5G technologies, REM, and PEM.

Full mobility of all MANET nodes and terrain variability, where the network operates, makes applying the ray-tracing method (RTM) [21] for its optimization and analysis challenging. A good solution for REMs is using measurement methods [22,23]. Modern transceivers are mainly based on SDR technology [24,25]. On the other hand, the possibility of designing radio waveforms allows for the implementation of an additional sensing (i.e., spectrum monitoring) process [26,27]. These relatively simple methods of empirical measurements will enable the building of electromagnetic situation awareness [23,27,28]. However, the results obtained refer only to the area where MANET is currently operating. Thus, these solutions are not sufficient to design the network in new terrain and assess the range capabilities of its nodes. Hence, the search for new solutions of this type is significant, especially in the military MANETs.

Different propagation models can be used to create REM. They can be classified into two main groups, i.e., statistical models and deterministic models [20]. We propose using PAMs, which can be the basis for determining coverage maps for network nodes. As shown in [28,29,30,31], the determined PAM can also be used in the process of analyzing the location of sensors to monitor the electromagnetic situation. The idea of the PAM was presented in [32], but the mechanisms of their generation were not described. In this paper, we focus on the detailed description of the novel PAM algorithm that was initially outlined in [33]. The proposed solution is based on three elements:

- determining the attenuation for given terrain profiles on specific azimuths outgoing radially from the transmitter (TX);
- using the PEM [34,35,36] for attenuation calculation along with the profiles;
- using linear interpolation to determine the attenuation values between the profiles to determine the entire PAM.

According to the best knowledge of the authors, the proposed approach can be considered original. The choice of PEM and linear interpolation resulted from the limited computing power of the devices on which REM will be implemented. In the general case, these methods may also be replaced by others, which provide similar abilities.

The basic PEM approach allows considering the terrain topography (with or without building heights), the electrical ground, and air-refraction parameters for determining propagation attenuation (i.e., path loss). In this paper, we present the novel PAM creation algorithm. To the best of the authors’ knowledge, it is an innovative and original solution. Typically, the PEM is used to determine the attenuation along a given propagation direction related to a specific terrain profile as a function of height above ground level. We show how to use the PEM at a given height of the receiving antenna to determine the attenuation on the surface.

The remainder of the paper is organized as follows. Section 2 provides a brief overview of the REM. The idea, short description of PEM, and algorithm of the proposed PAM approach with exemplary results are shown in Section 3. In Section 4, we analyze the efficiency of creating the PAM as a function of its dimensions. Next, in Section 5, an evaluation of the PAM accuracy versus angular resolution of terrain profiles is shown. A summary of the paper is presented in Section 6.

## 2. Radio Environment Maps

The REMs are a way of representing complex and multi-domain information for cognitive radio. By sharing and disseminating information contained in the REM, it is possible to provide cognitive functionalities such as situational awareness, deducing, learning, planning, and decision-making support. Consequently, the REM can be a database distributed among secondary users, centralized, or in a hierarchical topology where the central database interacts with local databases. REM are multi-domain databases containing terrain information, sensor data, simulation results of propagation models, regulations, and policies. PAMs are used in the planning of MANETs (also as cognitive radio networks) to increase the efficiency of such a network [37]. Using the measurements anywhere in the network area is impractical. Therefore, as part of the REM, spectrum measurements from available radio network nodes are collected. Then, data fusion is performed, i.e., available measurements are converted to estimate the level of interference in other, unexplored locations. The REM contributes to the development of cognitive mechanisms and the building of long-term knowledge. The REM introduces environmental awareness that is more difficult to achieve by the individual capabilities of individual network nodes. Therefore, the REM enables network nodes to be transformed into smart ones [22,23]. REM facilitates the adaptation of the radio network to a new environment. Therefore, the REM is a promising concept for the efficient operation of a cognitive network without overloading the cognitive node and can be connected to a network-based cognitive engine. Due to the REM dissemination, an individual secondary user can get to know the radio environment much more than is the case with the limited sensing possibilities. In this case, it is possible to use cooperative techniques to interact with primary users, avoid the hidden nodes problem, and increase the entire system’s efficiency. Due to its architecture, the REM can be broadly divided into global (G-REM) and local (L-REM). The REM complexity depends on the number of nodes and channels, the network coverage area, and the information granularity. REM synchronization is crucial for the accuracy and reliability of the information provided. Data must be effectively disseminated to avoid information that is obsolete or out-of-date.

The basic functionality of the REM is to build a dynamic interference map for selected frequencies in every interesting place. For this purpose, maps are used that represent the coverage of a given area with a radio signal, called radio-frequency (RF) layers of the REM, or shortly RF-REM [19]. For example, it is achieved by spectrum measurements obtained from sensors (i.e., network nodes) at specific locations. Since it is impractical and often impossible to perform measurements anywhere in the area of network operation, the available measurements are used to estimate the level of disturbances in other locations. Another approach is to use propagation damping prediction methods. It is a universal solution that does not depend on the current location of the sensor and allows you to analyze the area in which the network did not work before. Hence, the use of different propagation models for this purpose is common practice. The methods of creating the RF-REM can be divided into three main categories [19]:

- direct methods based on the interpolation approach, using spatial statistics and measurements of signal levels made in specific locations, make a direct estimation of the missing data (e.g., nearest neighbor, inverse distance weighting (IDW), and kriging);
- indirect methods based on the use of the TX location and its parameters as well as propagation models;
- hybrid methods combining both of the above approaches.

The above groups of methods have their advantages and disadvantages and are characterized by different computational complexity. The selection of an appropriate method is also dependent on the availability of specific data, e.g., the TX location and parameters, measurement results of signal levels in selected locations, variability of parameters over time, etc. Propagation methods allow for the creation of more accurate RF-REMs [38]. The developed solution based on the PEM can be used in indirect methods of creating the RF-REM.

## 3. Propagation Attenuation Map

PAMs show the path loss values relative to the defined point, usually located in the center. This part presents the proposed algorithm for PAM creation. It includes the general idea, algorithm view, and exemplary results.

### 3.1. Idea of PAM Algorithm

The main idea of the proposed PAM is based on the determination of a sparse matrix for given terrain profiles on specific azimuths outgoing radially from the TX. In the next step, the full PAM matrix is determined based on the interpolation method. We suggest using PAM in the first stage and linear interpolation—in the second stage. However, it should be emphasized that both the PEM and linear interpolation can be replaced by other methods that will allow for determining the full PAM matrix. Our choice resulted from the low computational expenditure of these methods.

**Figure 1:**
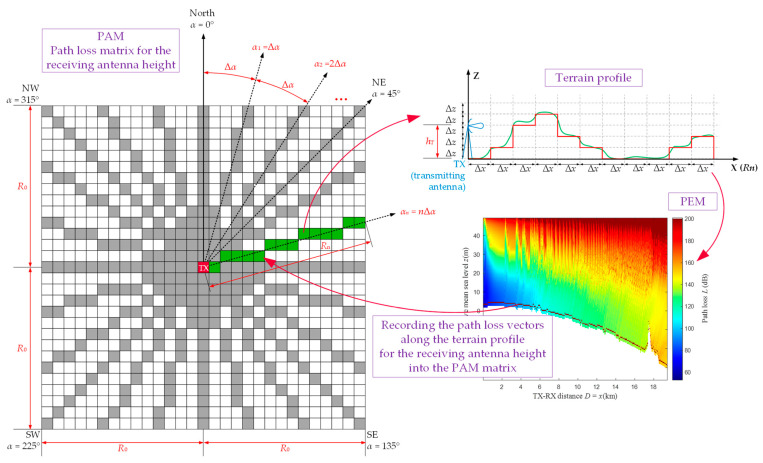
The idea of creating sparse PAM matrix.

**Figure 2:**
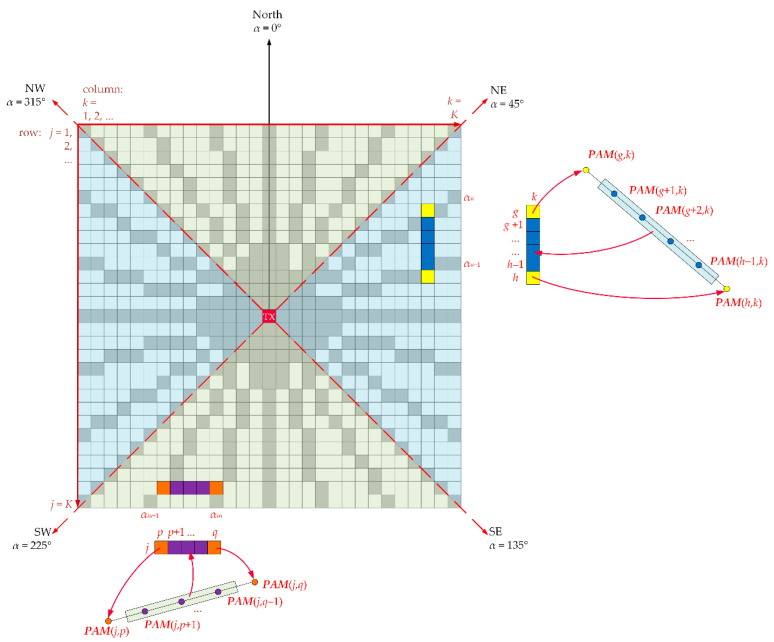
Interpolation method for creating full PAM matrix.

The main idea of the PAM algorithm is presented in Figure 1 and Figure 2. In this case, the PAM is identified with the path loss matrix, PAM, whose center cell corresponds to the TX location. In the first stage (see Figure 1), the so-called sparse PAM matrix is created based on the PEM for the terrain profile along a specific azimuth. In the second stage (see Figure 2), the sparse PAM matrix is completed based on interpolation of the non-zero values of the matrix cells. In this way, the final PAM is obtained, represented by the so-called full PAM matrix.

### 3.2. Parabolic Equation Method

For modeling tropospheric ducting propagation (TDP), the mode theory, RTM, and PEM are usually used. The first two methods require greater computational effort and have significant problems with modeling radio wave propagation in the troposphere, analysis of range-dependent environments, and higher frequency ranges. Moreover, the RTM does not allow for precise estimating of the field strength [36]. Hence, the PEM is widely used as an effective tool for modeling the TDP considering the terrain topography. Therefore, we used the PEM in the proposed PAM algorithm.

The development of the PEM and its widespread use took place in the early 1980s. However, the origins of the PEM as applied to the TDP modeling date back to 1946. Then, Leontovich and Fock [39] presented an analysis of TDP based on a solution to the parabolic equation. The first solutions were based on the first-order approximation of Taylor expansion, considering only the first two terms. This resulted in a low accuracy that allowed only analyzing a narrow beam in the elevation plane. This method was called narrow-angle PEM [36].

In 1973, Hardin and Tappert [40] proposed using the split-step Fourier transform (SSFT) algorithm to sequentially solve a parabolic equation along a given radius. This approach is one of the commonly used methods alongside two other numerical methods for solving a parabolic equation, i.e., a finite-difference method (FDM) and finite-element method (FEM).

From the TDP viewpoint, the PEM allows considering the terrain irregularity, the earth conductivity, and air refractivity for determining distributions of the electric field strength or signal attenuation. The phenomenon of air refraction is considered by using different refractivity profiles, which depend on changes in air parameters (i.e., temperature, pressure, and humidity) at different altitudes. The earth’s surface is also an important factor influencing the shape of these profiles, i.e., different nature of changes in the air refractivity index is above the surface of a sea, forest, mountains, desert, etc. [41]. This factor is also important in the analysis of the earth’s electrical parameters. These parameters are considered in the lower impedance boundary conditions for the parabolic equation [42,43]. The lower boundary conditions are applied at the contact level of the air and soil layers. In practice, when analyzing the TDP at greater distances, the earth’s curvature radius should be additionally taken into account [41].

Generally, the PEM is based on a numerical solution of the 2D parabolic equation in the Cartesian system:

(1) $$\frac{\partial^{2}}{\partial z^{2}}E\left(x,z\right)-2ik_{0}\frac{\partial }{\partial x}E\left(x,z\right)+k_{0}^{2}\left(n^{2}-1\right)E\left(x,z\right)=0,$$

where E(x,z) is electric field strength for the assumed radius—terrain profile (generalized x coordinate) and for any height (z coordinate), $i=\sqrt{-1},\;k_{0}=2\pi /\lambda$ is the wavenumber in the vacuum, λ is the wavelength, $n=\sqrt{\mu_{r}\varepsilon_{r}}$ is the refractivity index of air, $\mu_{r}$ and $\varepsilon_{r}$ are the relative magnetic and electric permeability of air, respectively.

Equation (1) is a parabolic approximation of the Helmholtz wave equation, whose full-wave solution is provided by the PEM [43]. This equation is solved numerically using one of the three mentioned methods (i.e., SSFT, FDM, or FEM) in a sequential manner from the TX along an analyzed terrain profile determined on a given azimuth direction of a propagation radius. Detailed descriptions of the algorithms are presented in [34,35]. A free FEM implementation in the MATLAB environment is also available [44,45].

We used the PEM implementation in MATLAB based on the SSFT approach, which considers the refractivity profile, terrain topography, impedance boundary conditions, and the earth curvature radius. In this case, we use the digital terrain elevation data (DTED) for determining the terrain profiles [46,47]. From the viewpoint of the proposed PAM solution, any PEM implementation can be used. Considering additional factors (e.g., the height of buildings, and vegetation) only influences the accuracy of the estimation of field strength or signal attenuation, but it does not matter for the implementation of our PAM algorithm.

Analyzing the literature, we see some similarities of the PAM algorithm to the 3D PEM approach presented in [48,49]. However, it should be highlighted that we do not determine the field strength distribution in the 3D space, but only 2D attenuation distribution (i.e., PAM) at the specific height of the receiving antenna. Such PAM as a heat map allows determining the radio coverage or the range of individual MANET nodes. In both cases, i.e., in the PAM and 3D PEM, radial rays from the TX (i.e., terrain profiles for which field distributions are determined) are applied. These rays are determined for specific azimuth directions with a given step.

### 3.3. PAM Algorithm

The proposed approach allows determining path loss values in all discrete points around a fixed TX position (marked in red in Figure 1). The indexes of individual pixels are correlated with geographic coordinates, and the distance between them depends on the available topography data, i.e., DTED resolution, ΔR. The analyzed area is contained in a square with a side length of $2R_{0},$ where $R_{0}$ means the radius of the analyzed terrain area for which the PAM is determined. The size of the created PAM matrix is K×K, where $K=\left\lfloor 2R_{0}/\Delta R\right\rfloor +1$. Calculations according to the PEM method are performed on defined directions around the TX depending on the angular resolution Δα.

An example procedure for determining attenuation on a selected azimuth direction $\alpha_{n}=n\Delta \alpha$ is depicted in Figure 1 (marked in green). The first step is to compute the terrain profile along the $\alpha_{n}$ direction. This profile contains the height of the terrain with Δz resolution for discrete distance steps (Δx) along $R_{n}=R_{0}/AF\left(\alpha_{n}\right),$ where $AF\left(\alpha_{n}\right)$ is the following angular factor: $AF\left(\alpha_{n}\right)=\sin\alpha_{n}$ for $45{}^{\circ}\;\leq \;\alpha_{n}\;\leq \;135{}^{\circ}$ or $225{}^{\circ}\;\leq \;\alpha_{n}\;\leq \;315{}^{\circ},$ and $AF\left(\alpha_{n}\right)=\cos\alpha_{n}$ for $0{}^{\circ}\;\leq \;\alpha_{n}\;<\;45{}^{\circ}$, $135{}^{\circ}\;<\;\alpha_{n}\;<\;225{}^{\circ},$ or $315{}^{\circ}\;<\;\alpha_{n}\;<\;360{}^{\circ}.$ PEM calculations based on the SSFT approach are performed in the next phase. Then, path loss vectors for a given height of the receiving antenna, $h_{R},$ are recorded into the PAM matrix. The method of determining the final PAM is explained in detail by Algorithms 1 and 2 for the sparse and full PAM matrices, respectively.

Algorithm 1 shows a determination way of the sparse PAM matrix based on PEM calculations. The procedure for determining the path loss values should be repeated for every defined azimuth and the corresponding terrain profile. Depending on the selected azimuth resolution, the number of loop executions (lines 4 to 10 in the algorithm) vary. **Algorithm 1***(Creating sparse PAM matrix based on PEM for selected terrain profiles)***Require:** DTED, DTED resolution (Δ*R*), radius of the analyzed area (*R*_0_), TX location, angular resolution (Δ*α*), receiving antenna height (*h_R_*).
 1.Calculate *K* = [2*R*_0_/Δ*R*] + 1, i.e., dimension of PAM matrix. 2.Create zero matrix for PAM with dimension *K* × *K*. 3.Set *n* = 0.
 **repeat**  4.Set *α_n_* = *n*Δ*α*.  5.Calculate *R_n_* = *R*_0_/*AF*(α_n_).  6.Determine terrain profile **TP**(*x*), vector of length *R_n_*, for TX location and azimuth *α_n_* based on DTED.  7.Calculate PEM matrix, **PEM**(*x*,*z*), for terrain profile **TP**(*x*), e.g., using SSFT method.  8.Read path loss profile **L**(*D*) = **PEM**(*x* = *D*, *z* = *h_R_*) from determined PEM matrix for receiving antenna height *h_R_*.  9.Save path loss profile **L**(*D*) to appropriate cells (green ones in Figure 1) of PAM matrix, **PAM**(*j*, *k*) = **PAM**(*α_n_*) = **L**(*D*), corresponding to analyzed azimuth α_n_, where *j*, *k* = 1, 2, …, *K* are indexes of PAM matrix and TX is located in central cell of PAM matrix (red one in Figure 1).  10.Set *n* = *n* + 1.
 **until**
*n*Δ*α* > 360°.
 11.Output **PAM** as sparse matrix based on PEM for selected terrain profiles.

The full PAM matrix is obtained by launching Algorithm 2. The proposed approach allows determining empty spaces (i.e., zero value cells) in the sparse PAM matrix (white ones in Figure 1). For this aim, we use linear regression. The interpolation method for creating the full PAM is explained in Figure 2. The input for this procedure is a partially completed path loss matrix, i.e., the sparse PAM matrix. Attenuation values in gray-marked points (see Figure 1 or Figure 2) are calculated according to Algorithm 1 described above. Zero cells (white ones in Figure 1) of the sparse PAM matrix are calculated based on linear regression and the two attenuation values located in the nearest non-zero cells. Depending on the coordinates of the considered point, i.e., indexes of matrix cell, there are two possible ways of linear interpolation:

- in a column—for the azimuth directions:
  ○ $225{}^{\circ}\leq \alpha_{n}\leq 315{}^{\circ}$ (i.e., k≤j≤K−k+1 for k=1,2,…,⌊K/2⌋+1),
  ○ $45{}^{\circ}\leq \alpha_{n}\leq 135{}^{\circ}$ (i.e., K−k+1≤j≤k for k=⌊K/2⌋+2,⌊K/2⌋+3,…,K),

- or in a row—for the remaining azimuth directions:
  ○ $315{}^{\circ}<\alpha_{n}<360{}^{\circ}$ or $0{}^{\circ}\leq \alpha_{n}<45{}^{\circ}$ (i.e., j+1≤k≤K−1 for j=1,2,…,⌊K/2⌋+1),
  ○ $135{}^{\circ}<\alpha_{n}<225{}^{\circ}$ (i.e., K−j+2≤k≤j−1 for j=⌊K/2⌋+2,⌊K/2⌋+3,…,K).

**Algorithm 2***(Creating full PAM matrix based on interpolation)***Require:** sparse PAM matrix (**PAM**).
 *Interpolation in columns*: 1.Set *k* = 1.
 **repeat**  2.Find *g*th row (*g* ≥ *k*) corresponding first non-zero cell in *k*th column (see one of yellow cells in Figure 2).
   **repeat**   3.Find *h*th row corresponding next non-zero cell in *k*th column (see one of yellow cells in Figure 2).   4.Calculate all zero cells in *k*th column between *g*th and *h*th rows, i.e., **PAM**(*j*, *k*) for *g* < *j* < *h*, based on Equation (2) (see blue cells in Figure 2).   5.Set *g* = *h*.
   **until**
*h* ≤ *K*–*k* + 1.  6.Set *k* = *k* + 1.
 **until**
*k* ≤ [*K*/2] + 1.
 **repeat**  7.Find gth row (*g* ≥ *K*–*k* + 1) corresponding first non-zero cell in *k*th column (see one of yellow cells in Figure 2).
   **repeat**   8.Find *h*th row corresponding next non-zero cell in *k*th column (see one of yellow cells in Figure 2).   9.Calculate all zero cells in *k*th column between *g*th and *h*th rows, i.e., **PAM**(*j*, *k*) for *g* < *j* < *h*, based on Equation (2) (see blue cells in Figure 2).   10.Set *g* = *h*.
   **until**
*h* ≤ *k*.  11.Set *k* = *k* + 1.
 **until**
*k* ≤ *K*.
 *Interpolation in rows*: 12.Set *j* = 1.
  **repeat**  13.Find pth column (*p* ≥ *j* + 1) corresponding first non-zero cell in *j*th row (see one of orange cells in Figure 2).
   **repeat**   14.Find *g*th column corresponding next non-zero cell in *j*th row (see one of orange cells in Figure 2).   15.Calculate all zero cells in *j*th row between *p*th and *q*th columns, i.e., **PAM**(*j*, *k*) for *p* < *k* < *q*, based on Equation (3) (see purple cells in Figure 2).   16.Set *p* = *q*.
   **until**
*q* ≤ *K*–*j*.
  17.Set *j* = *j* + 1.
 **until**
*j* ≤ [*K*/2] + 1.
 **repeat**  18.Find pth column (*p* ≥ *K*–j + 2) corresponding first non-zero cell in *j*th row (see purple cells in Figure 2).
   **repeat**   19.Find qth column corresponding next non-zero cell in jth row (see purple cells in Figure 2).   20.Calculate all zero cells in *j*th row between *p*th and *q*th columns, i.e., **PAM**(*j*, *k*) for *p* < *k* < *q*, based on Equation (3) (see purple cells in Figure 2).   21.Set *p* = *q*.
   **until**
*q* ≤ *j*–1.  22.Set *j* = *j* + 1.
 **until**
*j* ≤ *K*. 23.Output **PAM** as full matrix (without zero cells) based on interpolation.

In Figure 2, we showed these selected interpolation cases by colored cells of the PAM matrix. For column interpolation, the blue-marked zero cell values are determined based on two yellow non-zero PAM cell values of the sparse PAM matrix, i.e., PAM(g,k) and PAM(h,k) obtained for the azimuths $\alpha_{n}$ and $\alpha_{n+1},$ respectively. For row interpolation, the purple-marked zero cell values are derived from the two orange non-zero cell values of the sparse PAM matrix, i.e., PAM(j,p) and PAM(j,q) obtained for the azimuths $\alpha_{m+1}$ and $\alpha_{m},$ respectively. In these cases, the linear regression is performed according to the following formulas:

(2) $$\begin{array}{ccc} PAM\left(j,k\right)=\frac{PAM\left(h,k\right)-PAM\left(g,k\right)}{h-g}\left(j-g\right)+PAM\left(g,k\right) & \mathrm{for} & g<j<h \end{array},$$

(3) $$\begin{array}{ccc} PAM\left(j,k\right)=\frac{PAM\left(j,p\right)-PAM\left(j,q\right)}{q-p}\left(k-p\right)+PAM\left(j,p\right) & \mathrm{for} & p<k<q \end{array}.$$

According to Algorithm 2 presented above, the interpolation procedure is repeated column-by-column and then row-by-row so as to determine the path loss corresponding to the receiving antenna height at all points (i.e., cells) of the PAM matrix.

### 3.4. Exemplary Results

**Figure 3:**
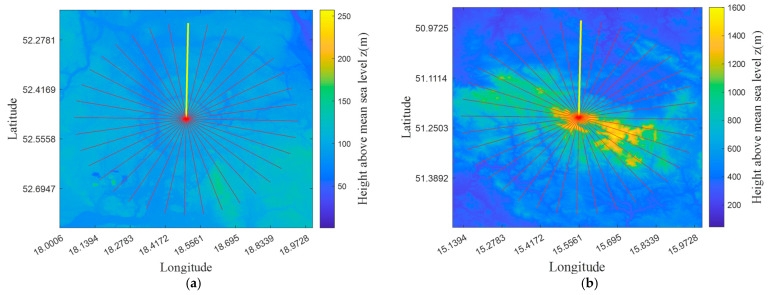
DTED-based elevation maps for two selected TX locations in (**a**) lowland and (**b**) hilly areas for which terrain profiles are calculated.

To check the developed algorithms, two positions of the TX have been proposed (see Figure 3). One of them is located in a lowland area (52.51 °N, 18.48 °E), the other–in a hilly terrain (51.22 °N, 15.55 °E). For each transmitting point located in the center of the map in Figure 3, terrain profiles are determined for the propagation directions (i.e., azimuths), which are shown by rays marked in red. In this case, the elevation maps obtained based on DTED2 [46,47], i.e., with an average resolution ΔR=30 m, are illustrated in the background.

**Figure 4:**
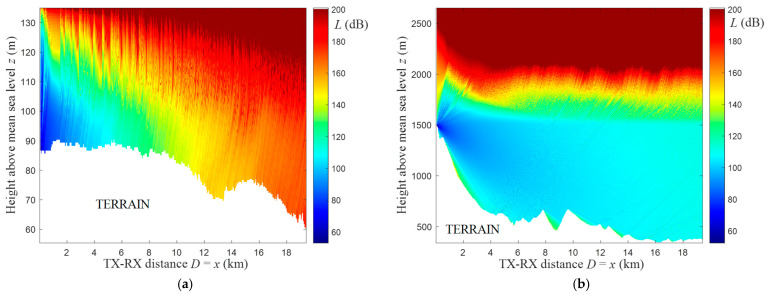
PEM-based electromagnetic field distributions for exemplary terrain profiles from (**a**) lowland and (**b**) hilly areas.

**Figure 5:**
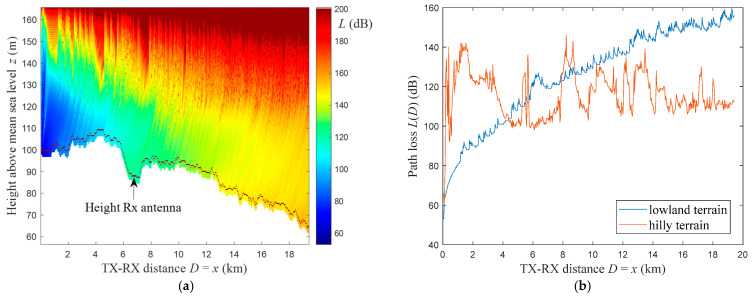
(**a**) Level of receiving antenna height marked above exemplary lowland terrain profile and (**b**) path losses versus terrain profile radius for analyzed receiving antenna height and calculated field distributions from Figure 4.

Figure 4 presents PEM-based electromagnetic field distributions obtained for yellow rays (see Figure 3) representing the terrain profiles at the azimuth $\alpha_{0}=0{}^{\circ}$ in two analyzed areas. In this case, the terrain profiles are shown as white shapes below the field distributions. In Figure 5a, we depict the exemplary level equal to the receiving antenna height, $h_{R}=2\;m,$ above the analyzed lowland terrain profile TP(x) from Figure 4a. The path loss read from the determined field distributions at the receiving antenna height above the terrain profile is the basis of the created PAM matrix. Figure 5b presents exemplary path losses versus terrain profile radius (i.e., length) for the two analyzed profiles and field distributions from Figure 4. 

**Figure 6:**
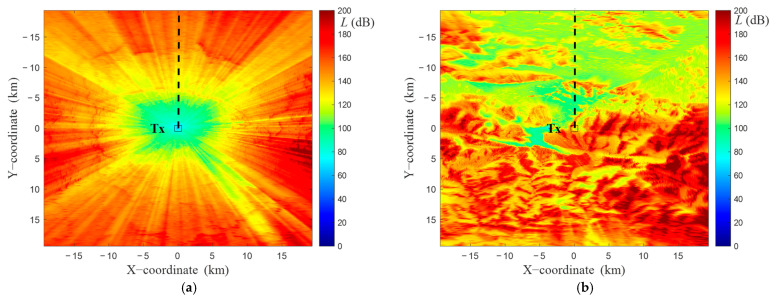
PAMs for two selected Tx locations in (**a**) lowland and (**b**) hilly areas.

The path loss values versus terrain profile radius, L(D), for analyzed receiving antenna height, $h_{R},$ and calculated field distributions (e.g., see Figure 5b) are written into the PAM matrix in the cells corresponding to the analyzed azimuth, $\alpha_{n}.$ In this way, the sparse PAM matrix, PAM(j,k), is created. It is the basis for determining the full PAM matrix by applying the interpolation process described in Algorithm 2. The final result of the developed algorithms is illustrated in Figure 6. In this case, we show PAMs (i.e., full matrices) for two selected TX locations (see Figure 3) in lowland and hilly areas, respectively. These PAMs were obtained based on 360 terrain profiles with a step Δα=1°. The PAM quality depends on the density of profiles on the basis of which it is generated. On the other hand, we may see the differentiation of the obtained PAMs depending on the terrain and TX position for which they are determined.

## 4. Efficiency of Creating PAM versus Its Dimensions

This part of the paper is devoted to studying the effectiveness of creating PAMs according to the previously presented algorithms. First, the metrics are defined, and then the research results are presented.

### 4.1. Metrics

As seen in the analyzed areas, there are points (i.e., sparse PAM matrix cells) for which calculations have not been made. Path loss values in these locations must be interpolated according to Algorithm 2. On the other hand, there are points (i.e., PAM cells), where calculations were performed repeatedly (more than once). This is due to the high density of the profiles near the TX. Let us introduce the redundancy parameter r as the number of path loss value calculations at a given point. As a consequence, it is possible to define the calculated points ratio CPR for the analyzed area

(4) $$CPR\left(\%\right)=\frac{CP}{TP}\cdot 100,$$

where CP and TP are the number of calculated points (with r≥1) and total number of points at the analyzed area, respectively.

To determine the number of all calculations performed, the redundancy values for all points in the analyzed area should be summed up

(5) $$CN=\sum_{s=1}^{TP}r_{s},$$

where s is the point index and $r_{s}$ is the redundancy value for a specified point s.

The CP measure introduced earlier considers the number of points, where the calculations were made at least once (i.e., r≥1). By correlating this parameter with the redundancy r, it is possible to define a new $CP_{r}$ measure meaning the number of points where the calculations were made exactly r times. Therefore, the previously introduced CN metric can also be expressed as

(6) $$CN=\sum_{r=1}^{r_{\max}}r\cdot CP_{r},$$

where $r_{\max}$ is the maximum value of r for the analyzed area.

The normalized number of points where the calculations were made exactly r times can be defined as follows:

(7) $$NCP_{r}\left(\%\right)=\frac{CP_{r}}{CP}\cdot 100.$$

Additionally, for evaluating the PAM creation efficiency, we use a computation time CT expressed in seconds. The measure considers the execution of all algorithm steps presented in Section 4.2, excluding the calculation of terrain profiles. The absolute values of this measure depend on the processing power of the used computer. Therefore, this parameter is used to compare different variants, e.g., different sizes of the analyzed area.

### 4.2. Result Analysis

**Figure 7:**
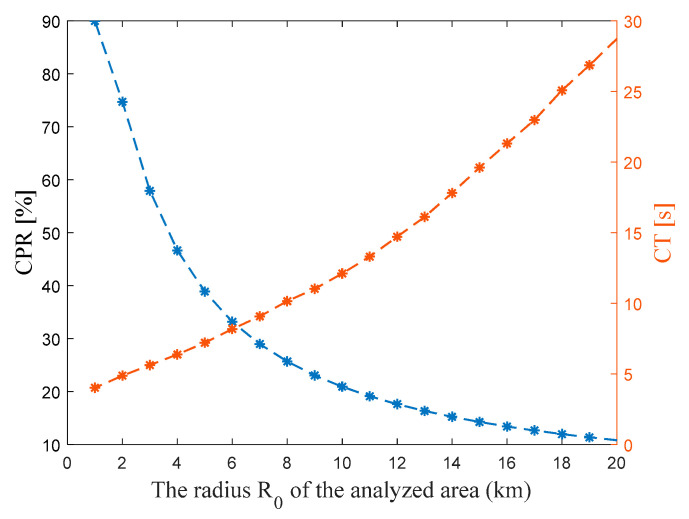
Calculated points ratio *CPR* and computation time *CT* for different radius *R*_0_.

The results presented in this part of the paper relate to studies carried out for various sizes of the analyzed area (square with a side length of $2R_{0}$). The angular resolution Δα for all tests are equal to 1°. Figure 7 shows the effect of increasing the radius $R_{0}$ on the calculated points ratio (i.e., CPR) values (blue color) and the calculation time (i.e., CT) needed to obtain the results (orange color). 

**Figure 8:**
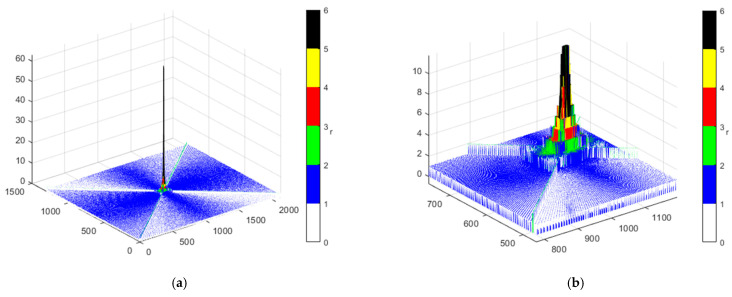
Redundancy *r* for points on sparse PAM matrix illustrated for (**a**) whole map area and (**b**) area with highest number of calculations.

As mentioned in the previous section, the CT metric depends on the processing power and is used for comparison purposes. The greater the value of the radius $R_{0},$ the longer the time needed to perform the calculations. For example, in the case of a tenfold increase in $R_{0}$ (from 2 to 20 km), the CT increases about six times. In the case of analyzing small areas, the vast majority of points on the PAM are calculated based on PEM (some points even multiple times, as shown in the following figures–see Figure 8). The larger the area of the analysis, the *CPR* value significantly decreases, and more points must be determined using the interpolation method presented in Figure 2. In the case of the radius $R_{0}$ equal to 1 km, about 90% of the points on the PAM are determined based on calculations. Increasing $R_{0}$ to 10 km results in a decrease in CPR to the level of only about 21%. In this case, 79% of points must be interpolated.

Figure 8 depicts the effect of duplicate calculation of points in generating the final PAM. In particular, the greatest redundancy of calculation is for the area next to the TX. Therefore, the increase in the number of calculations (i.e., CN) does not translate proportionally to the increase in CPR.

**Figure 9:**
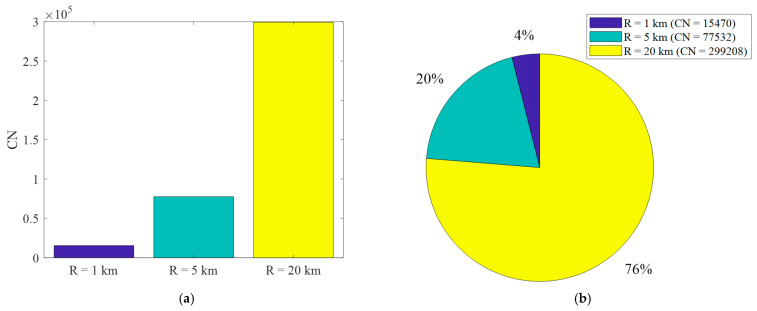
(**a**) Absolute and (**b**) normalized number of all calculations *CN* for different distances between TX and RX.

Another metric, independent of the processing power of the used computer, is the total number of calculations (i.e., CN). The absolute value of this measure for three distances $R_{0}$ is presented in Figure 9a. The normalized values of the calculations for these three cases are shown in Figure 9b.

**Figure 10:**
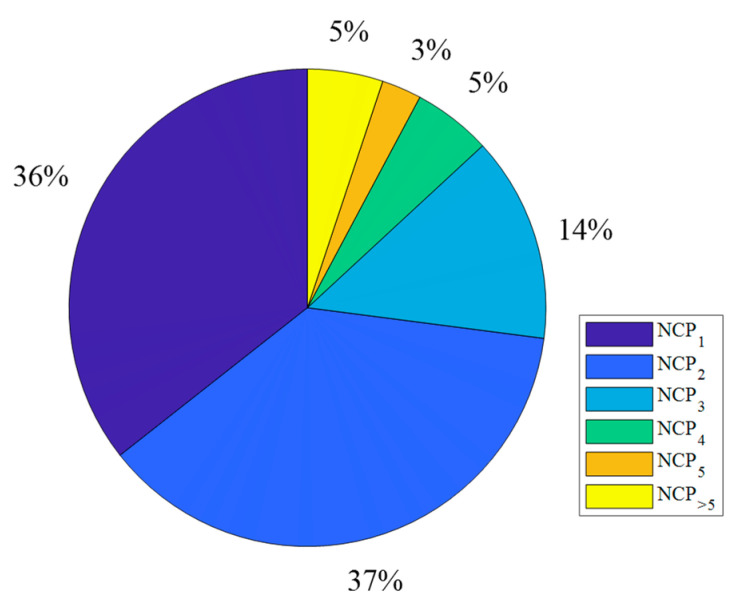
Normalized number of points where calculations were made exactly *r* times for TX-RX distance equals to 1 km.

Figure 10 shows the normalized number of points on the map for which the attenuation calculations are repetitive. In this case, the parameters $NCP_{2}$ and $NCP_{3}$ mean that 37% and 14% of points are calculated two or three times, respectively. On the other hand, $NCP_{>5}$ means the sum of points on the map is calculated 6 or more times. For PAM with a larger radius, i.e., $R_{0}=5\;\mathrm{km},$ the number of points without redundancy ($NCP_{1}$) is 92% and and the number of points without redundancy ($NCP_{1}$) is 97%.

## 5. PAM Accuracy Evaluation versus Angular Resolution of Terrain Profiles

**Figure 11:**
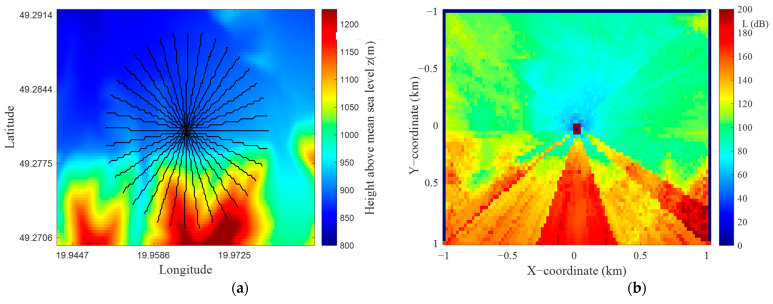
(**a**) DTED-based elevation map for selected TX location, (**b**) reference PAM for angular resolution Δα = 1°.

**Figure 12:**
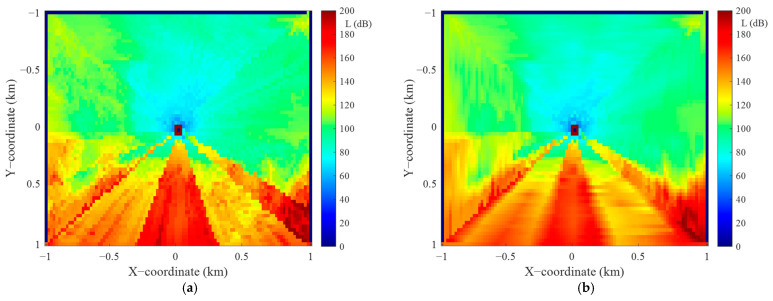
PAMs for selected angular resolutions (**a**) Δα = 2° and (**b**) Δα = 10°.

In this section, we present a preliminary assessment of the accuracy of the proposed algorithm versus the angular resolution of the terrain profiles. This assessment was made for the selected TX location with the coordinates (49.28°N, 19.96°E) and $R_{0}=1\;\mathrm{km}.$ The terrain around TX is shown in Figure 11a, while Figure 11b illustrates the PAM obtained for the angular resolution of the terrain profiles equal to Δα=1°. In our analysis, this PAM is used as a reference to determine the algorithm errors at another angular resolution of the terrain profiles. Figure 12 shows exemplary PAMs for the analyzed area and two different angular resolutions equal to 2° and 10°.

**Figure 13:**
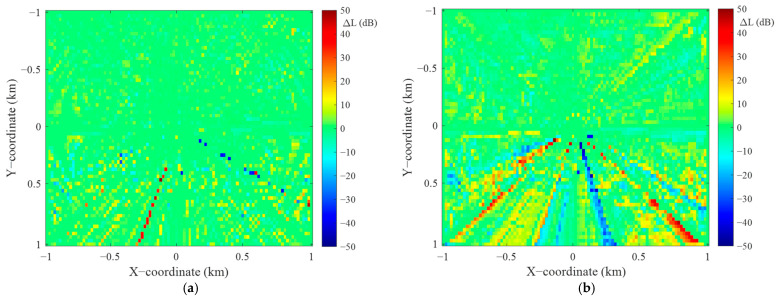
Error maps for selected angular resolutions (**a**) Δα = 2° and (**b**) Δα = 10°.

In the following, for every map point, PAM(j,k), we calculate the attenuation error

(8) $$\Delta L\left(j,k\right)=PAM_{\Delta \alpha }\left(j,k\right)-PAM_{\Delta \alpha =1{}^{\circ}}\left(j,k\right),$$

where $PAM_{\Delta \alpha }\left(j,k\right)$ and $PAM_{\Delta \alpha =1{}^{\circ}}\left(j,k\right)$ are the PAM values (i.e., attenuations) obtained for the analyzed Δα and reference Δα=1° angular resolutions of terrain profiles. In this way, the error maps, i.e., attenuation error matrices, are determined. Figure 13 shows sample error maps that were obtained for the two PAMs illustrated in Figure 12.

**Figure 14:**
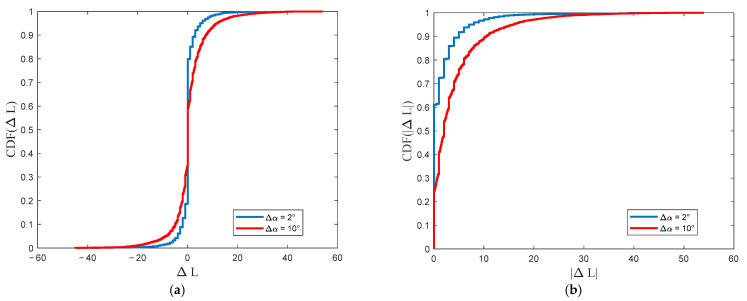
CDFs of (**a**) attenuation error and (**b**) attenuation error module for selected angular resolution of terrain profiles.

These error maps are the basis for determining the empirical cumulative distribution function (CDF), CDF(ΔL), and root-mean-square error (RMSE), RMSE(ΔL), of the attenuation error. Exemplary CDFs for the two analyzed error maps (see Figure 13) are presented in Figure 14a. Generally, we can judge that the obtained functions are symmetrical about the sign of ΔL. This means that the attenuation errors in the PAM with Δα oscillate around the attenuations for the reference PAM. Hence, we also computed the CDFs for the module of the attenuation error, CDF(|ΔL|), which are depicted in Figure 14b.

The CDFs analysis shows that increasing Δα causes a significant increase in the attenuation errors in determined PAMs. This is due to the smaller number of terrain profiles included in the calculations. For Δα=2°, 80% of the absolute attenuation errors are less than 3 dB, while for Δα=10°, it is 7 dB. For CDF(|ΔL|)=0.9, |ΔL|=5 dB and |ΔL|=10 dB for the angular resolutions equal to 2° and 10°, respectively.

Therefore, we may assume that for small Da, the number and time of calculations can be reduced at the expense of the accuracy of the PAMs obtained. This approach can be used in the preliminary stage of PAM determination, which requires initial visualization of the results. In the next step, with the platform computing resources available, the exact PAMs can be determined for the lower angular resolution of terrain profiles. 

**Figure 15:**
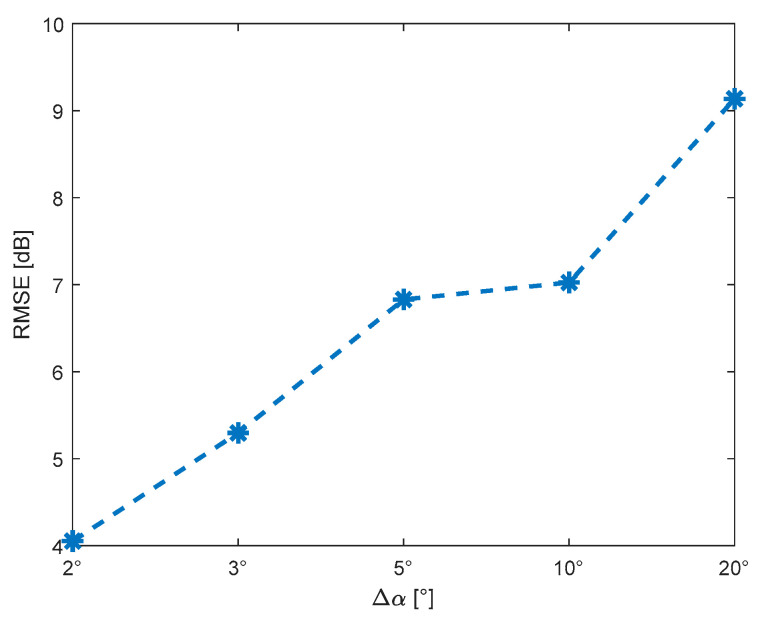
RMSE of attenuation error for selected angular resolution of terrain profiles.

Similar conclusions might be drawn by analyzing the RMSE scalar measure. Figure 15 shows the RMSE of attenuation error for selected angular resolution.

For the angular resolutions equal to 2° and 10°, we obtained RMSE(ΔL)=4 dB and RMSE(ΔL)=7 dB, respectively. Moreover, the graph shows that the error difference for Δα=5° and Δα=10° is small. In this case, we can obtain almost unchanged PAM accuracy with a two-fold reduction in the number of terrain profiles and a two-fold reduction in computation time.

The accuracy of PAM generation depends not only on the angular resolution of the profiles but also on their length (i.e., $R_{0}$), i.e., the PAM matrix dimension. A more detailed analysis of the PAM accuracy will be presented in our next work.

## 6. Conclusions

This paper focuses on a novel method of creating PAM as a crucial REM element, which allows for determining the ranges of radio network nodes. Our solution is based on the PAM determination in two stages. In the first stage, we determined the so-called sparse matrix for profiles outcoming radially from the TX. In the second stage, we used the interpolation method to calculate the full PAM matrix. The proposed approach is based on the DTED-based terrain profiles, PEM, and linear interpolation algorithm. Thereby, the developed solution considers the influence of topography on path loss calculation. The proposed algorithm was implemented in the REM for determining the radio ranges of tactical MANET nodes in the emerging military communication system. On the other hand, the proposed two-step approach to the PAM can be considered universal. This means that DTED, PEM, and linear interpolation can be replaced by others, which provide calculating the full matrix.

In this paper, we have presented a detailed description of the PAM generation algorithm based on the PEM for a given terrain topography. Metrics for evaluating the algorithm effectiveness depending on the PAM size have been defined. They were the basis for the assessment of the proposed solution for various PAM. Analyzing the results presented in the paper, it can be concluded that in the $R_{0}$ range up to about 3 km, over 50% of points in the PAM are calculated according to PEM. In the case of larger areas, it should be remembered that most of the points are interpolated, which affects the accuracy of determining the path loss values. These errors will get bigger with increasing distance from the TX. On the other hand, we presented a preliminary analysis of the PAM accuracy versus the angular resolution of the terrain profiles. The obtained results show that increasing Δα reduces the accuracy of determining the attenuation. However, with a two-fold increase in Δα, the computation time decreases twice, and the mean estimation error is equal to 4 dB. The obtained results also showed that increasing Δα from 5° to 10° only slightly increases the deviation of ΔL. The utilization of larger Δα can be used in the initial stage of PAM determination for the preliminary visualization of the results. In the next step, the exact PAMs may be determined for the lower angular resolution of terrain profiles.

Future work on PAM development will focus on assessing the impact of profile density on the path loss estimation accuracy, PAM utilization to evaluate the radio range of nodes (i.e., network coverage), comparison of the linear interpolation method with others (e.g., IDW or kriging), and PAM algorithm modification for the TX directional antennas.

## Data Availability

The data presented in this study are available on request from the corresponding author. The data are not publicly available due to project restrictions.

## Abbreviations

2D	two-dimensional
3D	three-dimensional
5G	fifth-generation
BMS	battlefield management system
CDF	cumulative distribution function
DSA	dynamic spectrum access
DTED	digital terrain elevation data
FDM	finite-difference method
FEM	finite-element method
G-REM	global radio environment map
IDW	inverse distance weighting
ISO	International Organization for Standardization
L-REM	local radio environment map
LTE	Long Term Evolution
LTE-A	Long Term Evolution Advance
MANET	mobile ad-hoc network
MIMO	multiple-input-multiple-output
NR	New Radio
OSI	open systems interconnection
PAM	propagation attenuation map
PEM	parabolic equation method
REM	radio environment map
RF	radio-frequency
RF-REM	radio-frequency radio environment map
RRM	radio resource management
RTM	ray-tracing method
RX	receiver
SDR	software-defined radio
SON	self-organizing network
SSFT	split-step Fourier transform
TX	transmitter
